# Does the IASP definition of pain need updating?

**DOI:** 10.1097/PR9.0000000000000777

**Published:** 2019-08-13

**Authors:** Murat Aydede

**Affiliations:** Department of Philosophy, University of British Columbia, Vancouver, BC, Canada

**Keywords:** IASP definition of pain, Taxonomy

## Abstract

The current IASP definition of pain has come under renewed criticisms recently. There is a new momentum for its revision as reflected by the fact that IASP has now a Presidential Task Force dedicated to look into whether there is enough warrant to update the definition. I critically review all the major criticisms of the current definition in detail, and raise new difficulties rarely discussed before. I show that none of the major criticisms has enough warrant to force us to substantially revise the current definition. Combined with the discussion of the new difficulties, there is nonetheless a need to restate the definition using slightly different terminology that will make the original intent of the current definition clearer and more precise. A restatement of the definition is proposed and its potential is discussed in light of some empirical questions that remain.

## 1. Introduction

The IASP definition of pain has not changed since its first publication in 1979.^12,25,29^ It has survived various criticisms raised in mid-to-late 1990s initiated by Anand and Craig.^5^ Similar criticisms and defenses continue to be voiced.^6,11,16–18,26,36,39,41–43,46^ Williams and Craig^47^ have recently raised the volume considerably with new criticisms and offered an alternative definition. Cohen et al.^14^ have also expressed their unhappiness with the current IASP definition and offered their own alternative. These then have gathered various reactions and produced counterresponses.^2,3,7,19,20,33–35,44,45,48–50^ As a result, IASP has formed a Presidential Task Force just to look into whether there is indeed any warrant to update the current definition. This work is still underway.

It is important to critically evaluate the criticisms directed at the IASP definition if we want to assess correctly whether there is any need to update it. In what follows, I will go over the major criticisms directed at this definition and assess their merit. My hope is that a rigorous critical review will be helpful to the members of the current Task Force and will prove useful for the profession at large by stimulating further critical and careful thinking that is much needed at this point in time.

## 2. Does the IASP definition require self-report?

Perhaps, the most influential and widely discussed criticism was the one raised by Anand and Craig^5^ in 1996:

“In its present form, however, the definition of pain challenges our understanding of pain because it does not apply to living organisms that are incapable of self-report. This includes newborn and older infants, small children, mentally retarded, comatose, demented, or verbally handicapped individuals, and all primate and non-primate animals.” (1996: 3)

As recently as 2017, Anand^4^ continued to press for this very complaint:

“This definition requires patients to describe their pain, by default establishing the primacy of self-report as a ‘gold standard.’ Although widely accepted across all healthcare professions and biomedical disciplines, this definition lacks applicability to non-verbal populations and ignores the cognitive and social dimensions of pain.” (2017: 1438)

As well, Anand here joins Williams and Craig^47^ in faulting the current IASP definition for not including the “cognitive and social” components of pain:

“First, acknowledging only sensory and emotional features excludes major and clinically important characteristics, in particular, cognitive, and social components. These components are often considered to be characteristic of chronic pain and can be overlooked in understanding acute pain, despite much evidence to the contrary.” (Williams and Craig 2016: 2421)

I will begin by discussing the charge that the IASP definition “does not apply to living organisms that are incapable of self-report.” Despite receiving a lot of attention and generating heated debate, it is important to understand that this criticism is based on a simple logical fallacy—a fallacy of confusing an or-statement (a disjunction) with an and-statement (a conjunction). Why this has not been noticed by the parties to the debate before is a historical and sociological curiosity. (Wright^49^ points out this too). Let me explain.

The current definition has the form of a disjunction:

“An unpleasant sensory and emotional experience associated with actual or potential tissue damage, or described in terms of such damage. (IASP 1979/2011)”

Let us make the form explicit by rewriting it explicitly in disjunctive form and expanding the determiner “such”:

A pain is:

(1) an unpleasant sensory and emotional experience associated with actual or potential tissue damage,

OR

(2) an unpleasant sensory and emotional experience described in terms of actual or potential tissue damage.

Please note that this rewriting is simply the result of making the logical structure of the formulation explicit. So, there should be no controversy about this. The form of the definition is *inclusive disjunction* of the form: “*p* or *q* (or both).” Such a disjunction is true if, and only if, at least one disjunct is true—false otherwise. (Each individual proposition making up a disjunction is called a “disjunct.”) So, in the above definition, each disjunct by itself provides a sufficient (but not a necessary) condition for something to be a pain. The necessary condition for something to be a pain is provided by the inclusive disjunction itself: the truth of [(1) *or* (2)] is both necessary and sufficient for something to be a pain. This is what it means for the formulation to provide a *taxonomic definition* for pain. Such a definition of pain is *in*correct if it fails to collect *all* and *only* those things that are intuitively pains: it fails if there are pains that are not captured by [(1) *or* (2)]. It also fails if there are things that satisfy [(1) *or* (2)] but are intuitively not pains. It is important to emphasize that the IASP definition is a taxonomic definition of *pain*, not just of *severe* or *clinically problematic pain*: it is supposed to cover *all* and *only* those things we correctly call “pains”—however slight or intense and however transient or persistent they may be.

It must be obvious that according to the IASP definition, *all* individuals who satisfy (1) count as having a pain, whether these individuals are “newborn and older infants, small children, mentally retarded, comatose, demented, or verbally handicapped individuals” or indeed whether they are “primate and nonprimate animals.” But obviously, clause (1) does *not* require any capability of self-report including linguistic report. There is simply no part of (1) that makes it applicable only to subjects that are capable of self-report. It is clearly not part of (1) that only those who can actually and verbally associate their experiences with tissue damage are capable of pain. The point of the use of “associated” will be explained below. But, here I merely point out that interpreting (1) as requiring actual verbal association by the subjects would be patently absurd, uncharitable, and unfair, bordering on the intentional misrepresentation. No verbal act of actual association by the agent is required by (1). I take this to be self-evident. This simple fact by itself refutes the claim made by Anand, Williams, and Craig, and by all the participants in the debate who assumed the same (and there were a lot of them even when they were critical).

On the face of it, Anand, Williams, and Craig think that the term “described” used in the second disjunct makes the *whole* definition not applicable to nonverbal individuals. But this is a mistake—a mistake of treating a disjunction as a conjunction (a conjunction is an and-statement, where each individual conjunct needs to be true for the conjunction as a whole to be true).

There is a *further* issue about whether the use of “described” in (2) does in fact make (2) *itself* inapplicable to nonverbal individuals as assumed by Anand, Williams, and Craig. This is by no means obvious either, and I will get back to it below. But let me first briefly inquire into why Anand, Williams, and Craig, and others were so easily misled to make this mistake. As is well known, the *Note* to the definition contains the following passage:^25^

“Many people report pain in the absence of tissue damage or any likely pathophysiological cause; usually this happens for psychological reasons. There is usually no way to distinguish their experience from that due to tissue damage if we take the subjective report. If they regard their experience as pain and if they report it in the same ways as pain caused by tissue damage, it should be accepted as pain.” (IASP 1979/2011)

The wish to properly handle the concern that motivated this passage (and the second disjunct in the main definition) was one of the most significant driving forces of the IASP definition. The resulting definition was radical and progressive precisely because it was geared to kill a widespread but narrow biomedical conception of pain. According to this conception, patients with chronic pain with no identifiable pathophysiological cause must have somehow “all made it up in their mind,” and therefore their pain is not real but must somehow be the product of their overactive imagination. As a result, these patients were likely to be ignored or denied proper care and treatment by the medical community. According to many, this problem has somewhat diminished (partly thanks to IASP's persistent campaign) but still exists today.

The last sentence in the quoted passage says (of the patient with chronic pain without any identifiable pathophysiological cause) that “if they regard their experience as pain and if they report it in the same ways as pain caused by tissue damage, it should be accepted as pain.” The crucial thing to notice is that it is somewhat easy to get the faulty impression that this sentence implies that “if they *do not* (or, *cannot*) report it in the same ways as pain caused by tissue damage, then it should *not* be accepted as pain.” I suspect that Anand and Craig (and others who followed suit in their footsteps) did think that this is indeed the implication of the *Note*, and this is probably why they thought that the IASP definition requires self-report. But if so, this is another logical fallacy: a conditional statement of the form ‘if *p* then *q*’ does not imply “if not-*p* then not-*q*.” This is to say that it would be a mistake to interpret the passage in the *Note* as implying that if there are individuals who cannot report or communicate their pains, their experiences are not pain. Not only is this inference *not* logically warranted by the passage, it would also be quite uncharitable to read it as implying this. The passage is simply concerned about those “many people [who] report pain in the absence of tissue damage or any likely pathophysiological cause.” There is no suggestion of any sort in the passage about those patients who do not or cannot report their pain—the passage is simply not talking about the nonverbal. To interpret this as implying that the passage is making a positive claim about the nonverbal individual is patently not warranted.

The need to add (2) to (1) in the form of a disjunction was prompted by the concerns expressed in the *Note*. There are many cases where an individual is in pain without any identifiable pathophysiology, which is to say, without any associated actual or potential tissue damage. These individuals would therefore *not* be covered by (1) alone. Hence, (2) comes into the definition by way of providing a disjunctive extension to (1): even in the absence of any associated actual or potential tissue damage (or any identifiable pathophysiology), the experiences of these individuals are genuine pain in so far as these unpleasant sensory and emotional experiences are *describable* in the same ways as those experiences in individuals with associated tissue damage. Describability does not require anybody's doing any actual description. The point of using “described” (or requiring describability) in the definition was to capture the idea that an unpleasant sensory and emotional experience is a genuine pain if, and only if, it shares the “usual sensory [and affective] qualities” of pain associated with actual or potential tissue damage. In other words, describability comes into the picture only as a proxy for phenomenological similarity—similarity to the collection of sensory and affective qualities distinctive of pain experiences associated with tissue damage. That this was the main idea (rather than actual linguistic description) is quite clear if we look at Merskey's^32^ own statement of it:

“It was evident that patients with psychological distress had experiences of pain in the body which resembled our experiences with physical illness, and both types of experience had to be regarded as pain, despite the lack of a physical cause for some of the former” (1994: S75).

So, the criticism and the persistent complaint that the current IASP definition of pain requires or implies self-report or that it does not apply to nonverbal organisms has no material foundation and is based on fallacious (and almost certainly, uncharitable) interpretation of the definition and the accompanying *Note*.

## 3. Does using “unpleasant” trivialize severe chronic pain?

Before turning to the question of whether the IASP definition neglects the cognitive or social components of pain, I want to briefly touch upon the second criticism of Williams and Craig:^47^

“Second, characterizing the experience simply as ‘unpleasant’ falls short: most acute or chronic clinically problematic pain is more than “unpleasant,” and the term potentially trivializes severe pain.” (2016: 2421)

This is one of the 3 major reasons that Williams and Craig give for updating the IASP definition. To address this, they propose to replace the term “unpleasant” with “distressing.”

The first thing to note is that the IASP definition is a definition of “pain,” not of “severe pain,” or not of “acute or chronic clinically problematic pain.” If you consider the entire history of the world, the majority of actual token pains that have ever occurred and will ever occur are almost certainly not “acute or chronic clinically problematic pains.” Indeed, think of the number of times on an average day that an average person hurts herself. A majority of these pains are not severe (luckily) and are not clinically important or problematic. How would you respond to someone (say, from NOAA) who objects that the definition of “wind” falls short because most tropical storms and hurricanes are more than “a current of air blowing from a particular direction” (dictionary.com)? I take it that IASP as an organization is in the business of understanding pain, not just “clinically problematic pain”—although the latter may be its primary focus for assessment, treatment, and health care policy purposes.

But more importantly, all sensory experiences produced by our sensory modalities come with a hedonic valence that can be modeled at the psychological level as a binary scale with continuous intensity values that vary from positive, passing through neutral, to negative. In other words, our sensations are always glossed with a hedonic/affective tone—negative or neutral or positive (this has been evident in affective neurosciences and emotion theory for quite some time^10,27,28^). English has a positive term (*pleasant*) that denotes the positive valence directly; but unfortunately, English has only a *negative* term obtained by negating the positive term (*unpleasant*) to denote the negative hedonic valence. Thus, the members of the original IASP Taxonomic Subcommittee did not have much choice in specifying *this* aspect of pain in a definition: the word “unpleasant” in the current definition is simply an umbrella term to state that pain is a negatively valenced sensory experience. It goes without saying, of course, that the range varies in intensity so that most clinically problematic pains tend to be quite severe. These are covered by the definition. Arguing against it on the ground that it trivializes severe pain misses the point of a taxonomic definition.

Nevertheless, we should be open to find better terms to express the fact that pains are negatively valenced experiences as far as their affective character is concerned.

## 4. Is pain social?

Another major complaint voiced by Anand, Williams, and Craig, and others is that the current IASP definition “…excludes major and clinically important characteristics, in particular, cognitive, and social components” (Williams and Craig 2016: 2421). Indeed, Williams and Craig^47^ propose the following definition to replace the current definition:

“Pain is a distressing experience associated with actual or potential tissue damage with sensory, emotional, cognitive, and social components.” (2016: 2420)

The claim seems to be that pain is *necessarily* cognitive and social, and by not mentioning these essential components or aspects of pain, the current IASP definition fails to provide necessary conditions for any experience to count as pain. Let us separate and restate these claims:

(C) Any pain experience is, by its essential constitution, cognitive.

(S) Any pain experience is, by its essential constitution, social.

Are (C) and (S) true? We cannot answer this question without understanding what these claims mean.

It is by no means clear what they mean. Note that we have a fairly clear idea about what it means for an experience to be sensory, unpleasant, or emotional. An experience is sensory only if it functions to detect and discriminate various energy forms (“adequate stimulus”) through relatively specialized neural systems. We can do psychophysical experiments to delineate the relevant phenomenological (sensory) quality space associated with sensory-discriminative information embedded in the experience. Similarly, a sensory experience is unpleasant only if it has a hedonic valence that functions in one's mental economy in whatever way the valence information is supposed to function. The hedonic valence of a sensory experience is usually manifest in one's experiential phenomenology and can be studied using psychophysical techniques. Emotions are more complex, but there is not much mystery about what it means for an experience to be emotional.

So, what does it mean for a sensory and affective experience to be *social*? Is sociality an essential phenomenological constituent in the way sensory and affective qualities are essential constituents in any pain experience? To the extent to which this question makes sense, the answer is “No.” Williams and Craig claim to present empirical evidence from the biopsychosocial modeling of pain phenomena. But as far as I can tell, all this evidence is evidence for the claim that social and cognitive (as well as psychological and biological) factors greatly *influence* pain experiences. I am pretty sure that this claim is true, and the research behind it is solid. But evidence for causal influence is not evidence for essential constitution. There are many other factors that causally influence pain experiences. For instance, there is now accumulating empirical evidence that weather conditions directly influence many types of pain.^1,13,40^ Suppose the causal influence of weather conditions can be systematically demonstrated to be a factor for how one experiences pain quite generally. Should we complain that the IASP definition neglects the *meteorological* component of pain experiences? Such a claim would border on the unintelligible: we have a good understanding of what it means for meteorological conditions to *influence* pain experiences, but we have no understanding of what it means for meteorological conditions to *be constituents* of a pain experience.

Scientific theorizing is all about causal mechanisms. Knowledge of causal mechanisms is empirical and quite crucial to manipulate outcomes in treatment and health care policy settings. But we should not confuse scientific theory construction and its application in clinical settings with the task of providing a taxonomic definition for the term “pain” that will collect all and only pains as intuitively understood by the scientists and the folk alike.

## 5. Is pain cognitive?

Evaluating claim (C) above is trickier. In the absence of a nontrivial working understanding of what “cognitive” means in this context, its extended discussion, I am afraid, will be controversial and not very useful. Still, I would like to make a few observations. First, note that “cognitive” does not mean “conscious.” It is relatively uncontroversial that we can have cognitive processes that are not conscious. Arguably, we can also have noncognitive processes that are conscious. Cognition has traditionally been compared and contrasted with sensation (or feelings). Having a homeostatic sensation of thirst or hunger, a gustatory sensation of saltiness, an olfactory sensation of a rose, a bodily sensation of pain or orgasm, etc., have all thought to be distinct from thinking, judging, remembering, imagining, belief acquisition, recognition, etc. The motivation for thinking that they are distinct is not that they do not influence each other, but rather that they are *differently constituted* and that they belong to different mental categories. The intuition is that a sensation (or a feeling) is a very different kind of thing than a thought or judgment. The standard way of cashing out this intuition has been that cognition involves the use and application of concepts, whereas having a mere sensation does not constitutionally necessitate having a concept. One can have a taste of something, thus have a conscious taste sensation, without being able to recognize or categorize what it is that one tastes, thus without being able to exercise any (nontrivial) concept applicable to what one tastes. This is not to say that expectations or perceived contextual factors will not have any influence on what one's sensation feels like, but it is to say that the having of a sensation is not the having of a (concept-involving) cognition. These are 2 different things, however, closely related to each other. Very few people would claim that the distinction between sensation, perception, and cognition is sharp and well delineated. Rather, it seems that there is a continuum of increasingly less analog information processing. Still, the lack of clear boundaries does not imply that there are no useful distinctions to be made between sensation, perception, and cognition. When we put the matter this way, the question becomes: where do we put pains along this continuum? Whatever the ultimate specific answer is, one thing seems clear to me: not in the cognition category. Hence, we should reject (C) above.

Anand, Williams, and Craig talk about the cognitive *component* or *dimension* of pain. Perhaps, they are thinking of pains as constituted by different elements: pains are constituted by both sensory and cognitive elements (among others). But what does this mean? What is the cognitive element such that *each and every* pain necessarily has it? If we accept that being cognitive implies concept involvement, then the capability of feeling pain will require the possession of some concepts. Apart from the difficulty of specifying what these concepts are, there is the real danger of overintellectualizing pain. We have animals, newborns, infants, cognitively incapacitated or handicapped organisms, or elderly with severe dementia. Intuitively, these groups seem capable of having pain but it is not clear at all whether they are capable of exercising the relevant range of concepts (whatever they are). It would be ironic to accuse Anand, Williams, and Craig of ignoring these populations—I am sure they have no such intentions (on the contrary!). But if the concept-involving interpretation of “cognitive” is not what they have in mind, what do they mean by it? They do not say. It seems to me that having observed the great efficacy of various forms of cognitive therapies for decades and the evidence coming from studies of pain modulation, they want to say something like: cognitive factors (what one believes, expects, anticipates, fears, hopes, wants, etc.) greatly influence pain experiences, hence pains are the kinds of mental phenomena that can be strongly influenced by cognitive factors. If this is all there is to their claim, I strongly agree with them. But this is no grounds to make the constitutive claim made by (C) above, and criticize the IASP definition for ignoring it. A lot of things causally influence a lot of other things. Detailing these influences is what scientific theories are for—a taxonomic definition is not a scientific theory.

## 6. What does “associated with” mean?

The current IASP definition does not have the difficulties and problems attributed to it by Anand, Williams, and Craig. Does it have other problems or difficulties? I will discuss 3 in this section and next. The first problem is with the term “associated with” used in (1). If we interpret it loosely in the British empiricist sense in which association is a matter of certain ideas following certain other ideas depending on the statistical correlations observed or past reinforcements, then it is not difficult to find counterexamples to the current definition. Take somebody who was traumatized when young by a dental surgery that went wrong. She now cannot stand the audiovisual presentation of a dentist drill—she finds her experience unpleasant. Take such an *audiovisual experience* of hers. This experience is sensory, unpleasant, and emotional associated with tissue damage: a clear case where (1) is satisfied. So, the current IASP definition *dictates* that this very audiovisual experience is pain. But clearly it is not. As a taxonomic definition, the satisfaction of [(1) or (2)] is meant to provide a sufficient (as well as necessary) condition for anything to be a pain. Clearly it does not do that, as things now stand. One might think that the definition can be tightened and made immune to this sort of counterexample by replacing “sensory” with “somatosensory.” But there can be somatosensory fear conditioning too—take somebody who is deaf and blind, but traumatized similarly. This person now cannot stand the somatosensory sensation of vibration associated with a dentist drill and the tissue damage it's caused. The problem these counterexamples point to is not with the word “sensory,” rather it is with the word “associated” understood loosely.

We can begin to see where the solution lies if we attend to the reasons for why the phrase was needed in the first place. Clearly, the 3 conditions for a pain experience—namely, being unpleasant, sensory, and emotional—are not jointly sufficient. What is needed is a criterion that would delineate the target sensations from all the rest that satisfy the 3 conditions. In standard psychophysical research, this is done by figuring out what the “adequate stimulus” is for the sensory system under investigation. For pain, the intuitive idea that needs to be captured is that pain sensations typically or paradigmatically arise out of noxious stimulation of bodily tissue that is apt to cause tissue damage. In other words, the term “associated with” was used to narrow down the class of unpleasant, sensory, and emotional experiences to only those that are in fact pain experiences on the basis of its adequate stimulus. The form of association here is the same to be found in the association between, say, the frequency of electromagnetic energy forms impinging on retinal cells and the resulting sensory colour experiences. If we interpret this phrase in the IASP definition in this way, the counterexample given above would be avoided: the audiovisual experience of a dentist drill would be excluded by the definition because this sort of experience does not *typically* or *paradigmatically* result from actual or potential tissue damage. In other words, we look at the kind of experience this particular experience is a member of, and ask if it paradigmatically results from noxious stimuli apt to cause tissue damage. The answer is “no”: the stimulus for it is not adequate stimulus for pain. If we keep this interpretation of “associated with” in mind when reading the current IASP definition, there is no need to revise the definition. If we want to bring clarity and precision and want to avoid misunderstanding, we may revise (1) explicitly in this way:

(1*) An unpleasant sensory and emotional experience that paradigmatically results from actual or potential tissue damage.

I realize that the term “paradigmatically” may sound a bit technical. Words like “typically” or “normally” can probably serve the same intended function, but I will stick with “paradigmatically” for present purposes because it makes the intended reading clearer.

Above I have mentioned that the term “described” in (2) was meant in the “describable” sense and that no act of actual description by anybody was intended. I suggested that the introduction of this clause was needed because (1) did not secure a necessary condition for an experience to be pain—although it was sufficient. There are many genuine pain experiences that come about in the absence of any identifiable potential or actual tissue damage. The intuitive idea was that these experiences can be brought into the scope of the definition by the disjunctive addition of (2). (2) on its own provides a sufficient condition for pain. The overall intent, then, was that the disjunction of (1) and (2), [(1) or (2)], would provide both necessary and sufficient condition for any experience to count as pain.

I have also suggested that describability gets into the picture merely as a reliable proxy for phenomenological similarity (similarity in terms of sensory and affective qualities)—as Merskey originally seems to have had in mind. If so, we can restate the second clause more explicitly and directly, bypassing the describability condition altogether:

(2*) An unpleasant sensory and emotional experience that is of the same kind or similar to an experience (that paradigmatically results from actual or potential tissue damage) in terms of its sensory and affective qualities.

Putting (1*) and (2*) together will give us a cleaner version of the IASP definition that I believe has been the intent behind the current formulation all along (see my 2017 for an extended argument to this effect):^7,8^

(IASP*) An unpleasant sensory and emotional experience that paradigmatically results from actual or potential tissue damage, or is of the same kind or similar to such an experience in terms of its sensory and affective qualities.

## 7. What do all pains share in common?

This reformulation of the current IASP definition is free of the problems raised and discussed so far. But it faces a new challenge. As far as I am aware, the difficulty and the problem associated with it was first raised by Howard Fields in an article published in 1999 in the context of a discussion of the affective dimension of pain:^7,9,23,49^

“By this official definition, it isn’t clear whether the unpleasant sensation reported by my nerve injury patients is pain, and in fact they often say it isn’t pain. Although there has been tissue damage, in that their nerve is injured, the words such patients use to describe the sensation do not correspond to stimuli associated with actual or potential tissue damage. This contrasts to the reported experience of patients with pains resulting from common tissue damaging insults. Such patients use words like ‘burning’, ‘cutting’ or ‘stabbing’ that evoke images of tissue being damaged. On the other hand, patients with nerve injury often report sensations that are unfamiliar and they use words that don't suggest threat or damage. Is their sensation pain even if they are uncomfortable using that word to describe it? Should we try to shoehorn their experience into categories that we have contrived?” (Fields 1999: S62)

Fields seems to raise 2 questions here that we need to identify and separate from each other. One is that the first clause in the definition may not be sufficient. Fields points out actual cases where many neuropathic pain patients seem to satisfy all the conditions laid out in (1) or (1*) but may still not be in pain as they describe their own experiences avoiding using the term “pain.” This is an important difficulty that points to a need to further clarify the relation between the experience and the tissue damage assumed in (1) or (1*). (1*) can be interpreted as covering any unpleasant sensory and emotional experience that results from *any* sort of actual or potential tissue damage—in which case Fields' patients are problem cases due to the nerve injuries they sustain.

The subtle but important point to make here to handle this difficulty is to pay attention to how “paradigmatically” is meant to function in the definition. The idea here is to fix the scope of experiences in (1*) to those that result from only those noxious stimuli that constitute “adequate stimuli,” ie, stimuli that stimulate nociceptors properly—in the way they are evolutionarily designed to function.^42^ We cannot use this technical vocabulary in the definition, but we can read “paradigmatically” in such a way as to exclude experiences that result from nerve injury or disease or dysfunction. In other words, the intent is to restrict the first clause, by this use of “paradigmatically,” to collect only nociceptive pain, in other words, only pains solely due to the normal functioning of the nociceptive mechanisms as these were biologically designed to function. If we do this, the patients Fields has in mind are no longer counterexamples to the sufficiency of (1*). On the other hand, to the extent to which these patients' experiences are pain (to the extent to which they are willing to use “pain” to describe their own experiences), they are so in virtue of their experiences' satisfying the second clause—just as it was intended. Neuropathic and nociplastic pains—in so far as they are pains—are genuinely pains in virtue of being phenomenologically similar to nociceptive pains. The idea is that despite their involving different mechanisms and aetiologies, they are all pains in virtue of having sufficient phenomenological similarity to a paradigm group. This is the upshot of the IASP definition as reformulated here and seems to capture intuitively well both the folk and clinical/scientific usage of the term “pain.”

However, Fields may be raising a second and deeper question, which should be faced seriously and squarely by any proposal that is similar to the IASP definition^7,23,49^: Are there unpleasant sensory experiences that are typically accepted in the clinical literature as pains (and are not due to known pathophysiology) that escape the basic intent of the IASP definition even under its proposed reformulation? Many forms of neuropathic and especially nociplastic pains (eg, experiences involved in fibromyalgia, complex regional pain syndrome, irritable bowel syndrome, and primary migraine) *may* have sensory and affective qualities that are not sufficiently similar to nociceptive pains. It may be possible to classify some of these experiences under dysesthesia. But how can we be sure that all pains, or rather all experiences commonly accepted as pains, are sufficiently phenomenologically similar to pains due to normal nociception?

This is a difficult question to answer. Part of the reason is the inherent subjectivity of pain experiences—the sensory and affective qualities of pain experiences (indeed of any experiences) are accessible only from a first-person point of view as long as we lack the means to determine what they are from an objective perspective. Nevertheless, the question is ultimately an empirical one: we need to empirically investigate the quality spaces within these experience kinds, and compare and contrast them to come up with a similarity matrix. We then need to determine how people use “pain” vis-à-vis this matrix to find out what quality subspace it carves out. Various psychophysical techniques combined with clinical experience can be used to tackle this question.^21,22,24,30,31^ There are also other techniques that attempt to combine the first-person and third-person methodologies.^37,38^

Note, however, that although the question is difficult and ultimately empirical, it need not be presently settled for definitional purposes. The definition of “pain” expressing our common semantic understanding of the term is not a theory and may involve exactly the same amount of vagueness there may be in the phenomenon itself. In some (hopefully rare) occasions, there may be no fact of the matter whether the phenomenological similarity demanded by the second clause is good enough and of the right kind for an experience to count as pain—certainly further complicated by lack of relevant descriptors. Even the patients or the subjects themselves may not be able to tell. If this is correct, it means that there is a certain amount of indeterminacy in the very nature of pain itself as we commonly understand it. But if this correct, we cannot capture the phenomenon with an on-and-off definition that will treat pain as an on-and-off phenomenon. A certain amount of indeterminacy in our definition, in other words, may be the correct thing to incorporate, and the IASP definition as reformulated does just that.

As things now stand, does anybody have any better answer to the question: in virtue of what do neuropathic and nociplastic pains count as pains? The IASP definition (as reformulated) seems to deliver the best answer: in virtue of sufficiently resembling standard nociceptive pains in crucial respects. This may not be *exactly* correct; but if not, we need to discover this empirically, and the results might be significant. Perhaps, we might find out, *some* experiences that have been standardly categorized under neuropathic, nociplastic, or idiopathic “pains” are not pains after all. Conversely, *some* unpleasant sensory experiences that have been usually categorized as not pain (eg, some intense itches, certain intensely unpleasant bodily sensations associated for instance with dizziness, vomiting, etc.) might turn out to be better categorized as pains. The IASP definition does not close these possibilities—on the contrary!

So, despite there being a lingering worry as to whether (IASP*) will accurately collect *all* persistent or chronic pain cases, especially due to neuropathy or dysfunction of the nociceptive system, the way to tackle the worry is to read the definition as open to (indeed, as encouraging) further empirical investigation. When clinicians and medical professionals talk about pain, they have in mind chronic pain, or more generally, pain as a health problem, as something that needs to be controlled, dealt with. Viewed from this perspective, anchoring the understanding of problematic pain in terms of similarity to acute nociceptive pain due to tissue damage seems to get things the wrong way around. Nevertheless, if pain is a natural kind such that acute, persistent, and chronic pains are subspecies, the question arises as to what makes them unified as pain. Phenomenological similarity of a certain sort seems like the right answer to this question—hence the IASP definition (see Corns^15^ for a sustained challenge to the claim that pain is a natural kind.) However, this worry should motivate further critical thinking about the need to separate the definition of chronic pain (or certain kinds of chronic pain) from the definition of pain.

## 8. Conclusion

In this work, I have defended the current IASP definition of “pain” against the criticisms raised by Anand, Williams, and Craig. I have argued that their criticisms do not warrant updating the IASP definition in any fundamental way. I have then raised 3 further criticisms.

First, the current formulation is vulnerable to counterexamples showing that the definition does not provide sufficiency conditions for pain, if “associated with” is read loosely. The remedy, I have suggested, is to force a reading of this phrase as given by (1*) above. I have also proposed that the definition should be formulated without using the term “described,” which was nothing but a dispensable proxy for phenomenological similarity in the first place. I believe that the original framers of the current definition had more or less this meaning in mind as captured by (IASP*) above. Thus, I take (IASP*) to be not an update in substance but a cleaner restatement of the original definition.

Second, I suggested that Fields' concern that (1) or (1*) may still not be sufficient can be handled by a certain reading of the term “paradigmatically” restricting the clause to standard nociceptive pains (this term may be replaced with “typically” or “normally” as long as we keep the intended interpretation in mind.)

Third, I pointed out that Fields' concern can be expressed as posing a general challenge to the definition itself in this reformulated version: Are all experiences regularly categorized under the label “pain” sufficiently similar to nociceptive pains in terms of their sensory and affective qualities? Conversely, are all experiences that are sufficiently similar to nociceptive pains standardly categorized under the label “pain”? These questions, I believe, are important empirical questions that cannot be settled from one's armchair. Further research is required, but as things now stand, the IASP definition is our best guide to a common semantic understanding of what the word “pain” means.

## Disclosures

The author has no conflict of interest to declare.
